# Supplementary material for: Content-rich biological network constructed by mining PubMed abstracts
Source: BMC Bioinformatics. 2004 Oct 8;5:147. doi: 10.1186/1471-2105-5-147 (PMC528731; doi:10.1186/1471-2105-5-147)
Supplement: Additional File 2 — The original results of the above study (non-essential files are deleted to keep the file size under the limit set by BMC bioinformatics). [file 1471-2105-5-147-S2.bz2 › chilibotAdditionalFile2/dip10/83ID8798570E297/html/EPHB1_EFNB1.html]

 


 **EPHB1** and **EFNB1** 
  
Found 51 abstracts in PubMed, retrieved 10.  
 

 What does Google say? 
 PDF only 
| .edu only 

---

**Interactive relationship** (e.g. stimulation, inhibition, etc)

**Neutral relationship**- EphB  [ **EPHB1** ]  receptors influence growth of ephrin B1  [ **EFNB1** ]  positive statoacoustic nerve fibers.  Ref: 12405963 Eur J Neurosci, 2002
- Our data suggest that branches of EphB  [ **EPHB1** ]  expressing axons are attracted medially by ephrin B1  [ **EFNB1** ] , and provide molecular mechanisms for D V mapping in visual centers.  Ref: 12165470 Neuron, 2002
- Alternatively, EphB  [ **EPHB1** ]  receptor signaling may act as a ligand density sensor and titrate signaling pathways that promote branch extension toward an optimal ephrin B1  [ **EFNB1** ]  concentration found at the TZ.  Ref: 12702655 Development, 2003

**Non-interactive relationship** (e.g. studied together, co-existance, homology, etc.)

- In vivo disruption of EphB  [ **EPHB1** ]  ephrin B  [ **EFNB1** ]  function by application of exogenous EphB  [ **EPHB1** ]  or expression of dominant negative ephrin B  [ **EFNB1** ]  ligand in dorsal retinal axons causes these axons to shift dorsally in the tectum, while misexpression of wild type ephrin B  [ **EFNB1** ]  in ventral axons causes them to shift ventrally.  Ref: 12165469 Neuron, 2002
- A major recent advance is the demonstration that EphB  [ **EPHB1** ]  receptor forward signaling and ephrin B  [ **EFNB1** ]  reverse signaling mediate axon attraction to control dorsal ventral retinal mapping along the lateral medial tectal axis.  Ref: 12593983 Curr Opin Neurobiol, 2003
- Ephrin B  [ **EFNB1** ]  and EphB  [ **EPHB1** ]  are distributed in matching dorsoventral gradients in the embryonic Xenopus visual system with retinal axons bearing high levels of ligand dorsal projecting to tectal regions with high receptor expression ventral.  Ref: 12165469 Neuron, 2002
- EphB  [ **EPHB1** ]  receptors are expressed in a low to high DV gradient by retinal ganglion cells RGCs, and ephrin B1  [ **EFNB1** ]  is expressed in a low to high LM gradient in the tectum.  Ref: 12702655 Development, 2003
- We propose that Xdsh, which is expressed in various tissues, is involved in EphB  [ **EPHB1** ]  and ephrin B  [ **EFNB1** ]  signaling related to regulation of cell repulsion via modification of RhoA activity.  Ref: 12574121 EMBO J, 2003
- In contrast, stimulation of Jurkat cells with the EphB  [ **EPHB1** ]  receptor ligand ephrin B1  [ **EFNB1** ]  does not cause Cbl phosphorylation.  Ref: 12794130 J Immunol, 2003
- We report that the EphB  [ **EPHB1** ]  receptor ligand, ephrin B1  [ **EFNB1** ] , may act bifunctionally as both a branch repellent and attractant to control the unique mechanisms in mapping the dorsal ventral DV retinal axis along the lateral medial LM axis of the optic tectum.  Ref: 12702655 Development, 2003
- Together, these results suggest a role for EphB  [ **EPHB1** ]  receptors in providing guidance signals to ephrin B1  [ **EFNB1** ]  positive SAG nerve fibers.  Ref: 12405963 Eur J Neurosci, 2002
- We conclude that in the intestinal epithelium beta catenin and TCF couple proliferation and differentiation to the sorting of cell populations through the EphB  [ **EPHB1** ]  ephrin B  [ **EFNB1** ]  system.  Ref: 12408869 Cell, 2002
